# Detection and Whole-Genome Characterization of a G8P[1] Group A Rotavirus Strain from Deer

**DOI:** 10.1128/genomeA.01339-16

**Published:** 2016-12-08

**Authors:** Srivishnupriya Anbalagan, Jessica Peterson

**Affiliations:** Newport Laboratories Inc., Worthington, Minnesota, USA

## Abstract

Rotavirus A strain 14-02218-2, with genome constellation G8P[1]-I2-R2-C2-M2-A3-N2-T6-E2-H3, was isolated from newborn fawns. The 14-02218-2 rotavirus strain is related to bovine and bovine-like rotavirus strains. To our knowledge, this is the first report on whole-genome-based characterization of a deer rotavirus G8P[1] strain.

## GENOME ANNOUNCEMENT

Rotaviruses are major enteric pathogens of humans and livestock animals, especially in young calves and piglets (1, 2). Clinical presentations range from asymptomatic infection to acute diarrhea that may lead to death due to severe dehydration or other complications (1, 2). Rotaviruses were isolated from primates, cows, pigs, deer, lamb, sheep, goats, camelids, horses, dogs, foals, mice, and birds (1–4).

In 2014, lung, heart, kidney, spleen, and, intestine samples were submitted for diagnostic testing from a farm where several newborn fawns were found dead within a few days of birth. Samples tested positive for rotavirus A (cycle threshold [*C_T_*] value, 22.81) by quantitative reverse transcription-PCR (RT-PCR). Rotavirus A isolation was attempted on African green monkey kidney cells (Marc-145) (ATCC CRL-2378.1) and inspected daily for cytopathic effect (CPE). If CPE was noted, the medium was collected and analyzed for rotavirus A by quantitative RT-PCR (data not shown). RNA sequencing was performed using Ion Torrent Personal Genome Machine (PGM) (Life Technologies, Grand Island, NY), as described by Anbalagan et al. (5). Sequence reads were assembled into contigs using the SeqMan NGen program (DNAstar, Madison, WI).

The lengths of open reading frames for VP1 to -4, VP6, VP7, and NSP1 to -5 were 3,267, 2,643, 2,508, 2,331, 1,194, 981, 1,476, 954, 942, 528, and 597 bp, respectively. The genes encoding VP1, VP2, VP3, VP4, VP6, VP7, NSP1, NSP2, NSP3, NSP4, and NSP5 of 14-02218-2 are closely related to RVA/Cow-tc/USA/WC3/1981/G6P[5] (96%), RVA/Cow-tc/USA/WC3/1981/G6P[5] (98%), RVA/Bovine-tc/USA/NCDV/1971/G6P[1] (95%), RVA/Bovine-tc/USA/NCDV/1971/G6P[1] (95%), RVA/Cow-wt/JPN/Tottori-SG/2013/G15P[14] (94%), RVA/Human-wt/US/2012841174/2012/G8P[14] (98%), RVA/Cow-tc/USA/WC3/1981/G6P[5] (97%), RVA/Simian-tc/USA/RRV/1975/G3P[3] (99%), RVA/Bovine-tc/SouthAfrica/OAgent/1965/G8P[1] (98%), RVA/Human-wt/GTM/2009726790/2009/G8P[14]  (99%), and RVA/Cow-wt/JPN/Tottori-SG/2013/G15P[14] (98%), respectively. A major difference in sequence identity (94%) was observed for VP6, encoding a major internal structural protein.

Strain 14-02218-2 possessed genotype constellation G8P[1]-I2-R2-C2-M2-A3-N2-T6-E2-H3. The genotype constellation of strain 14-02218-2 is similar to bovine DS-1-like strains, commonly found in rotavirus A strains from order Artiodactyla, which includes pigs, peccaries, hippopotamus, camel, deer, giraffe, pronghorn, antelopes, sheep, goats, and cattle. Intermingled distribution of deer and bovine herds over the same geographical location might be one of the reasons for the isolation of bovine-like G8P[1] from deer. This study provides important insights into the complete genetic makeup of a deer rotavirus strain and its genetic relatedness to rotavirus A from other host species.

### Accession number(s).

Sequences were submitted to GenBank under accession numbers KU962128 (VP1), KU962129 (VP2), KU962130 (VP3), KU962131 (VP4), KU962132 (VP6), KU962133 (VP7), KU962134 (NSP1), KU962135 (NSP2), KU962136 (NSP3), KU962137 (NSP4), and KU962138 (NSP5).
